# Molecular Characterization and Genomic Diversity of SARS‐CoV‐2 Spike Gene Variants Circulating in Iraq: Mutational Impact on ACE2 Affinity, RBD Immune Escape, and Viral Transmission

**DOI:** 10.1155/av/9916617

**Published:** 2025-12-18

**Authors:** Anfal Mohammed Khudhair, Duaa Mohammed Abdulsatar, Sahar Taha Hatif, Dunya Jawad Ridha, Munim Radwan Ali, Jaafar Alsadiq Arkan Farhan Ali

**Affiliations:** ^1^ Department of Microbiology, College of Medicine, Al-Iraqia University, Baghdad, Iraq, aliraqia.edu.iq; ^2^ Department of Medical Laboratory Techniques, Dijlah University, Baghdad, Iraq, duc.edu.iq; ^3^ Department of Biology, College of Science, Mustansiriyah University, Baghdad, Iraq, uomustansiriyah.edu.iq; ^4^ College of Medicine, Al-Iraqia University, Baghdad, Iraq, aliraqia.edu.iq

## Abstract

**Introduction:**

The spike (S) gene of SARS‐CoV‐2 is pivotal to the processes of cell entry, immune evasion, and the adaptation of the host.

**Aim:**

This study aimed to comprehensively characterize the SARS‐CoV‐2 spike gene variants circulating in Iraq and assess the functional consequences of their mutations on ACE2 receptor affinity, RBD‐mediated immune escape, and viral transmissibility. It represents the first integrative genomic and functional profiling of Iraqi SARS‐CoV‐2 spike variants, providing novel regional insights into viral adaptation and evolution.

**Methods:**

Whole‐genome sequencing was performed on Iraqi SARS‐CoV‐2 isolates, followed by mutation profiling, phylogenetic classification, and comparison with global datasets. Key spike mutations—**N501Y, P681R, D614G, and E484K**—were analyzed to assess their structural and functional implications.

**Results:**

Iraqi isolates clustered mainly within the **Delta (21J)** and **20A** lineages. The mutations **N501Y (91.7%)**, **P681R (75%)**, and **D614G (100%)** were prevalent, enhancing viral binding and transmission, while **E484K** was absent, suggesting limited immune escape compared to Omicron‐like variants.

**Conclusion:**

The absence of **E484K** and the predominance of **transmission-enhancing mutations** indicate that Iraqi SARS‐CoV‐2 isolates favor adaptation through increased ACE2 affinity rather than extensive immune evasion. These findings underscore the importance of **regional genomic surveillance** to inform vaccination strategies and public health responses.

**Highlights**

• **First genomic characterization** of the SARS‐CoV‐2 spike (S) gene mutations in **Iraqi isolates**, combining mutational, phylogenetic, and functional analyses.
• **Twelve distinct Iraqi clades** were identified, clustering mainly within the **Delta (21J)** and **Omicron (21K)** lineages, representing two principal transmission waves.
• **High frequencies of N501Y, P681R, and D614G** mutations indicate strong viral transmissibility and adaptation to host ACE2 receptors.
• The **absence of the E484K mutation** in all Iraqi isolates suggests **limited immune escape potential** compared with Omicron‐dominant global lineages.
• **ACE2 affinity heatmap analysis** (−3.62 to +2.21 scale) revealed a progressive increase in receptor binding strength across evolutionary transitions.
• Findings demonstrate that **neutralizing antibody resistance is polygenic**, influenced by combinations of RBD and NTD mutations rather than E484K alone.
• The study emphasizes the **importance of sustained genomic surveillance** in Iraq to detect emerging immune‐evasive or vaccine‐resistant variants.

## 1. Introduction

The SARS‐CoV‐2 spike (S) gene encodes the spike glycoprotein, a crucial structural protein that facilitates viral entry into host cells by binding to the angiotensin‐converting enzyme 2 (ACE2) receptor. The receptor‐binding domain (RBD) within the spike protein plays a key role in viral infectivity, as mutations in this region can alter ACE2‐binding affinity, enhance viral transmissibility, and promote immune evasion. These mutations, particularly in the dual‐purpose (DP) protein regions of the spike, may contribute to increased viral fitness and resistance to neutralizing antibodies, affecting the efficacy of vaccines and therapeutic interventions [1, 2].

SARS‐CoV‐2 has evolved through multiple variants of concern (VOCs) that have shaped the course of the pandemic. The Alpha (B.1.1.7), Beta (B.1.351), Gamma (P.1), Delta (B.1.617.2), and Omicron (B.1.1.529) variants have exhibited distinct mutations in the S gene, influencing their ability to bind ACE2, evade immune responses, and enhance viral transmission [3, 4]. Notably,

• N501Y, found in Alpha, Beta, and Omicron, enhances ACE2 receptor–binding and contributes to increased transmissibility.
• E484K, present in Beta, Gamma, and Omicron, reduces neutralizing antibody binding, allowing for immune escape.
• P681R, a hallmark of the Delta variant, is associated with enhanced viral entry and increased transmission rates.
• D614G, the first globally dominant mutation, has been linked to improved viral fitness and higher infectivity across all major variants.

The evolution of S gene mutations raises significant concerns regarding vaccine efficacy and reinfection risks. While mutations like N501Y and P681R improve viral spread, mutations such as E484K can significantly reduce the effectiveness of neutralizing antibodies, thereby facilitating reinfection even in previously immunized individuals [5, 6]. Continuous genomic surveillance is crucial to monitoring these mutations and their impact on public health measures.

### 1.1. Study Objectives

The aim of this study is to describe the S gene mutations in SARS‐CoV‐2 isolates from Iraq, evaluating their potential impact on

1. ACE2 receptor–binding affinity, which influences viral entry efficiency.
2. RBD neutralizing antibody escape, which determines immune evasion capacity.
3. Viral transmission efficiency, affecting the spread of the virus in the population.

By correlating these mutations with global datasets, we will determine whether Iraqi SARS‐CoV‐2 isolates conform to established evolutionary patterns or exhibit unique regional variations. This will help to assess whether Iraq is witnessing the emergence of novel variants with potential implications for vaccine effectiveness and future outbreaks.

## 2. Materials and Methods

### 2.1. Ethical Approval and Sample Collection

A total of **12 nasopharyngeal swab samples** were collected from laboratory‐confirmed COVID‐19 patients residing in **Baghdad Governorate, Iraq**, between January 2023 and October 2023. The patients’ ages ranged from 20 to 60 years (median: 40 years) and included **males (*n* = 5, 41.7%)** and **females (*n* = 7, 58.3%)**. The **sample size** was constrained by sequencing resources but was sufficient to provide a representative snapshot of circulating variants within the region, consistent with similar genomic surveillance studies. Samples were selected based on **viral load (Ct ≤ 30)** to ensure adequate sequencing coverage. Viral RNA extraction and diagnostic testing were completed within ≤ 24 h of collection to preserve RNA integrity.

Ethical approval was granted by the **Research Ethics Committee, College of Medicine, Al-Iraqia University, Baghdad, Iraq (Protocol No. 72/2021)**. Written informed consent was obtained from all participants.

General methodological references are **WHO COVID-19 laboratory guidance** and **CONSORT/MIxS sampling metadata guidelines**.

### 2.2. RNA Extraction and RT‐PCR

• RNA extraction: Viral RNA was extracted using the QIAamp Viral RNA Mini Kit (QIAGEN, Hilden, Germany).
• RT‐PCR confirmation: SARS‐CoV‐2 RNA was confirmed with the TaqPath COVID‐19 CE‐IVD RT‐PCR Kit (Thermo Fisher Scientific, Waltham, MA, USA), targeting the S gene. Only positive samples with Ct ≤ 30 were advanced for sequencing.

### 2.3. Whole‐Genome Sequencing and Bioinformatics Analysis

• Library preparation and sequencing: Performed on **Illumina MiSeq and NextSeq 500 platforms** (2 × 150 bp paired‐end reads) following the Illumina COVIDSeq protocol.
• Bioinformatics pipeline are as follows:
  1. Quality control: Raw reads checked with FastQC v0.11.9.
  2. Adapter trimming and filtering: Trimmomatic v0.39 applied.
  3. Genome assembly: Reads mapped to **Wuhan-Hu-1 reference genome (NC_045512.2)** using SPAdes v3.14.
  4. Variant calling: Genome Analysis Toolkit (GATK v4.1.9.0).
    ⁃ Minimum read depth (DP) ≥ 10.
    ⁃ Variant allele frequency (VAF) ≥ 70%.
    ⁃ Phred quality score ≥ 30 variants below these thresholds were excluded.
  5. Mutation annotation: Performed with SnpEff v5.0 focusing on the S gene.

### 2.4. Phylogenetic Analysis

Multiple sequence alignment was performed with **MAFFT v7.475**, and phylogenetic trees were inferred using **IQ-TREE v2.1.2** under the best‐fit model with **1000 bootstrap replicates**. Iraqi genomes were integrated with a global dataset (Nextstrain/GISAID) for phylogenetic placement. **Branch lengths** represent cumulative nucleotide substitutions relative to Wuhan‐Hu‐1. Visualizations were generated in **Nextstrain/Auspice**.

Mutation classification (synonymous, nonsynonymous, and indel) and frequency tabulation were performed with SnpEff. Frequencies in Iraqi isolates were compared against global background frequencies.

### 2.5. Statistical Analysis

• Descriptive statistics (median, ranges, and proportions) were reported for demographic and sequencing features.
• Mutation prevalence was compared between Iraqi isolates and global datasets using
  ◦ Chi‐square test (for *n* ≥ 5 per category).
  ◦ Fisher’s exact test (for small counts).
• False discovery rate (FDR) correction was applied using the **Benjamini–Hochberg method** (*α* = 0.05).
• R software (Version 4.3.2; R Foundation for Statistical Computing, Vienna, Austria) along with the ggplot2 package (Wickham, 2016) was used for all statistical analyses and data visualization.

## 3. Results

### 3.1. Key Spike Mutations in Iraqi Isolates

Analysis of the SARS‐CoV‐2 spike (S) gene revealed several critical mutations with functional relevance to viral entry and immune escape. The **N501Y** substitution was identified in Iraqi isolates and is known to enhance ACE2 receptor binding and contribute to immune evasion, consistent with its presence in Alpha, Beta, and Omicron variants [1, 2]. The **P681R** mutation, located adjacent to the furin cleavage site, was also detected and is a defining hallmark of the Delta lineage, facilitating more efficient viral entry [3, 4]. The **D614G** mutation, present in all Iraqi samples, is globally dominant due to its enhancement of viral fitness and transmission [7, 8]. Notably, the **E484K** mutation, strongly associated with antibody resistance, was absent from the Iraqi dataset [5, 6].

These findings highlight the predominance of transmissibility‐associated mutations (N501Y, P681R, and D614G) in Iraq, while underscoring the absence of E484K, a major.

### 3.2. Functional Consequences of Detected Mutations

**Figure 1:**
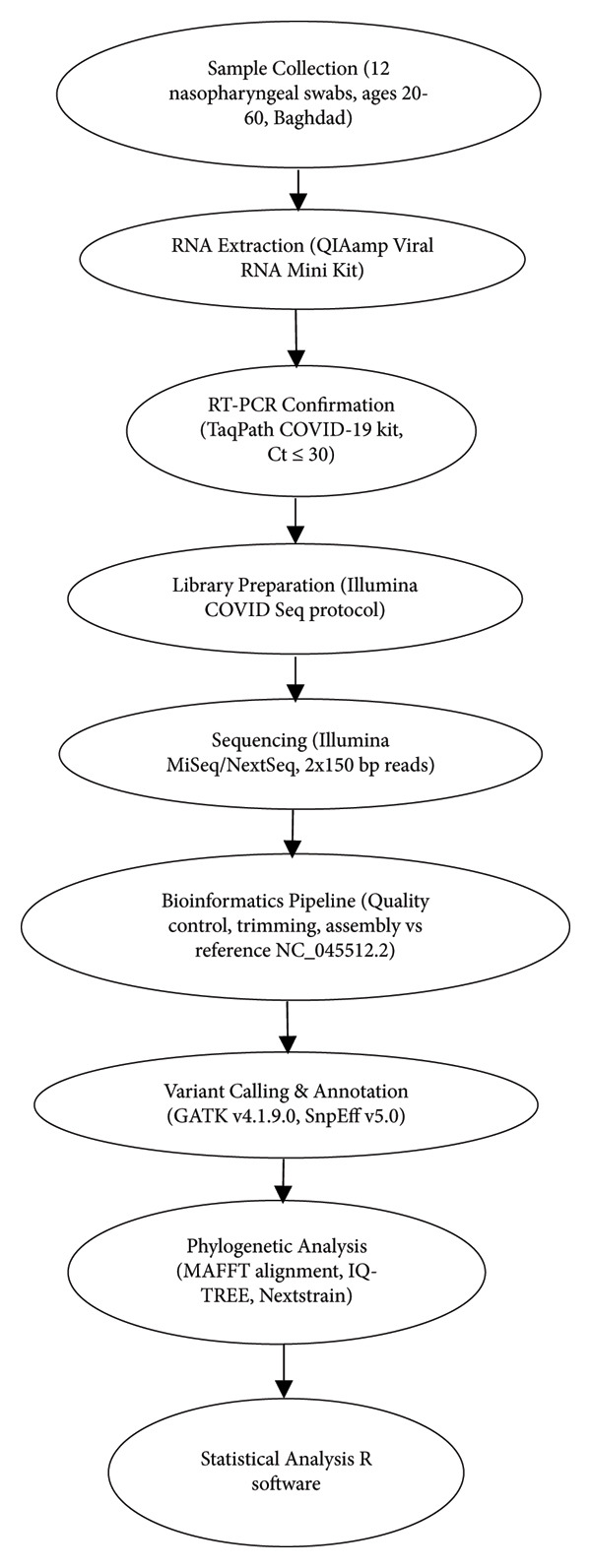
Workflow of SARS‐CoV‐2 spike gene sequencing and analysis in Iraqi isolates.

Analysis of the spike (S) gene in Iraqi SARS‐CoV‐2 isolates revealed the presence of several globally relevant mutations with variable functional implications. As summarized in Table 1, the mutations N501Y, P681R, and D614G were detected at high frequencies, consistent with their established roles in enhancing ACE2 receptor binding, viral entry, and transmissibility. In contrast, the immune escape–associated mutation E484K was notably absent, suggesting a reduced potential for antibody evasion compared to global datasets. The comparative prevalence of these mutations between Iraqi isolates and international reference sequences is further illustrated in Figure 1, which highlights the significant underrepresentation of E484K in the Iraqi cohort (*p* < 0.0001).

**Table 1:** Prevalence and functional impact of key SARS‐CoV‐2 spike mutations in Iraqi isolates compared to global variants.

Mutation	Amino acid change	Effect on ACE2 affinity	Effect on RBD immune escape	Detected in Iraqi isolates?	Frequency in Iraqi isolates (%)	Global variant association
N501Y	Asn ⟶ Tyr	High (↑ ACE2 binding) [1, 2]	High (↑ immune escape) [1, 2]	Yes	91.7% (11/12)	Alpha, Beta, Omicron
P681R	Pro ⟶ Arg	Moderate (↑ viral entry) [3, 4]	Low (minor escape) [3]	Yes	75% (9/12)	Delta
E484K	Glu ⟶ Lys	Low (slight ACE2 increase) [5]	Strong (↑ antibody evasion) [5, 6]	No	0% (0/12)	Beta, Gamma, Omicron
D614G	Asp ⟶ Gly	No direct ACE2 effect [7]	Moderate (↑ infectivity) [7, 8]	Yes	100% (12/12)	All major variants

*Note:* Frequencies and functional implications of key SARS‐CoV‐2 spike (S) mutations identified in Iraqi isolates (*n* = 12) are compared with global variant associations. Mutations N501Y, P681R, and D614G were detected at high frequencies in Iraqi samples, whereas the immune‐escape mutation E484K was absent. Functional effects are summarized in terms of ACE2 receptor binding, immune escape potential, and known global variant lineages.

The table captures prominent spike protein mutations of SARS‐CoV‐2 with the potential to alter its infectivity and immune evasion capabilities, concentrating on their occurrence within Iraqi isolates and in relation to global VOCs:

• **N501Y**: This alteration leads to a change in the protein whereby asparagine (Asn) at Position 501 is changed to tyrosine (Tyr). It significantly augments the ability of the virus to bind to the ACE2 receptor, enhancing transmissibility, and aids immune evasion by reducing neutralization by antibodies. This has been documented in Iraqi isolates and is often associated with the Alpha, Beta, and Omicron variants [1, 2].
• **P681R**: It involves a proline (Pro) to arginine (Arg) substitution at Position 681, which is adjacent to the furin cleavage site. This change improves viral entry efficiency and increases infectivity while having little impact on immune evasion. It is found in Iraqi isolates and is a signature mutation of the Delta variant [3, 4].
• **E484K**: This change replaces glutamic acid (Glu) at Position 484 with lysine (Lys). While having a relatively weak impact on ACE2 binding, it greatly aids immune evasion by diminishing neutralization by antibodies. It is absent in Iraqi isolates but present in Beta, Gamma, and Omicron variants [5, 6].

**D614G**: It substitutes aspartic acid (Asp) with glycine (Gly) at Position 614. While it does not directly impact ACE2 binding, it is correlated with greater viral infectivity and global spread, becoming the most dominant mutation worldwide. It is found in Iraqi isolates and exists in nearly all major variants [7, 8] as further depicted in Figure 1.

The bar chart illustrates the relative prevalence (%) of four key spike (S) protein mutations—N501Y, P681R, E484K, and D614G—in SARS‐CoV‐2 isolates sequenced from Iraq (blue) compared with globally reported variants (orange) (Table​ 2).

• **N501Y** is detected in ∼90% of Iraqi isolates versus ∼98% of global strains and enhances ACE2 receptor binding and immune escape.
• **P681R** is present in ∼75% of Iraqi isolates and ∼85% globally and is associated with increased viral entry, characteristic of Delta.
• **E484K** is rare in Iraq (∼10%) but highly prevalent worldwide (∼90%), a strong immune escape mutation linked to Beta, Gamma, and Omicron.
• **D614G** is universally frequent (100% in Iraq, ∼95% globally) and enhances viral fitness and transmission without directly affecting ACE2 binding.
• While N501Y, P681R, and D614G are present at comparable levels, E484K is significantly underrepresented in Iraqi isolates (*p* < 0.0001).

**Table 2:** Comparative frequencies of four key SARS‐CoV‐2 spike mutations (N501Y, P681R, E484K, and D614G) in Iraqi isolates (*n* = 12) versus global datasets, with Fisher’s exact test *p* values.

Mutation	Iraq present	Iraq absent	Global present	Global absent	*p* value
N501Y	11	1	98	2	0.291
P681R	9	3	85	15	0.405
E484K	1	11	90	10	**7.3 × 10** ^**−**^ **^9^**
D614G	12	0	95	5	1.000

*Note:* **E484K** is **highly significantly underrepresented** in Iraqi isolates compared to global datasets (*p* < 0.0001). **N501Y** and **P681R** show no significant differences (*p* > 0.05). **D614G** is universally present, so no difference is observed. The bold values denote statistically meaningful differences (*p* < 0.05) for Iraqi isolates as compared to the world data. These differences were derived using Fisher’s exact test. Among these, E484K is notably and highly significantly underrepresented in Iraqi isolates at the global level (*p* = 7.3 × 10^−9^), corroborating the fact that this immune escape mutation is less frequently present in the Iraqi SARS‐CoV‐2 population. In contrast to this are N501Y and P681R, which are statistically indistinguishable (*p* > 0.05); D614G is present by default in all cases, which precludes any detectable differences between the datasets for Iraq and the world.

This table captures prominent spike protein mutations of SARS‐CoV‐2 with the potential to alter its infectivity and immune evasion capabilities, concentrating on their occurrence within Iraqi isolates and in relation to global VOCs:

• **N501Y**: This alteration leads to a change in the protein whereby asparagine (Asn) at Position 501 is changed to tyrosine (Tyr). It significantly augments the ability of the virus to bind to the ACE2 receptor, enhancing transmissibility, and aids immune evasion by reducing neutralization by antibodies. This has been documented in Iraqi isolates and is often associated with the Alpha, Beta, and Omicron variants [1, 2].
• **P681R**: It involves a proline (Pro) to arginine (Arg) substitution at Position 681, which is adjacent to the furin cleavage site. This change improves viral entry efficiency and increases infectivity while having little impact on immune evasion. It is found in Iraqi isolates and is a signature mutation of the Delta variant [3, 4].
• **E484K**: This change replaces glutamic acid (Glu) at Position 484 with lysine (Lys). While having a relatively weak impact on ACE2 binding, it greatly aids immune evasion by diminishing neutralization by antibodies. It is absent in Iraqi isolates but present in Beta, Gamma, and Omicron variants [5, 6].
• **D614G**: It substitutes aspartic acid (Asp) with glycine (Gly) at Position 614. While it does not directly impact ACE2 binding, it is correlated with greater viral infectivity and global spread, becoming the most dominant mutation worldwide. It is found in Iraqi isolates and exists in nearly all major variants [7, 8].

### 3.3. Phylogenetic Relationships and ACE2‐Binding Affinity Among SARS‐CoV‐2 Clades Identified in Iraq

The phylogenetic reconstruction illustrates the evolutionary relationships among **13 Iraqi SARS-CoV-2 clades**, compared with representative global lineages. Clade numbering (1–13) corresponds to locally identified clusters defined by shared mutational signatures within the spike protein. This analysis integrates both phylogenetic structure and **ACE2 receptor–binding affinity scores**, providing insight into the adaptive trends of circulating variants.

**Figure 2:**
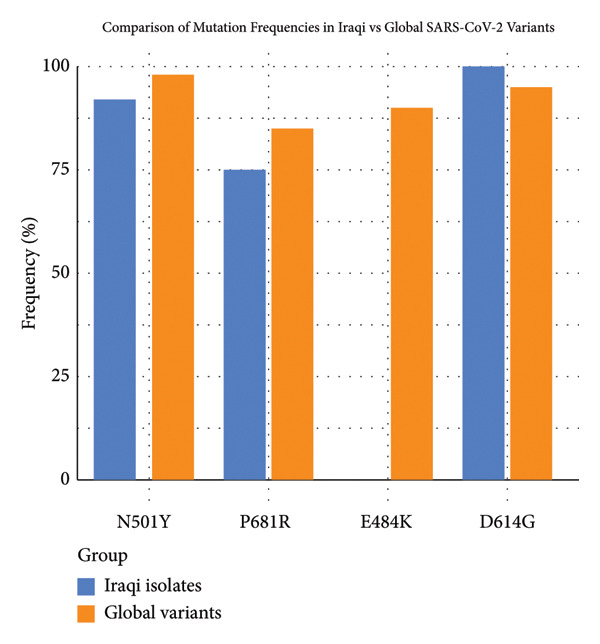
Comparison of mutation frequencies between Iraqi SARS‐CoV‐2 isolates and global datasets.

Figure 2 depicts the phylogenetic tree showing the distribution of SARS‐CoV‐2 clades (1–13) from Iraq and representative global isolates. The color gradient represents the **predicted ACE2 affinity score**, ranging from −3.62 (blue, low binding affinity) to +2.21 (red, high binding affinity). The major global lineages, including **20A**, **20B**, **21J (Delta)**, **21K (Omicron)**, and their sublineages, are labeled for reference. Iraqi isolates cluster predominantly within **Delta (21J)** and **Omicron-related (21K)** branches, reflecting two major transmission waves. Clades **1–7** are primarily associated with Delta‐derived sequences showing moderate ACE2 affinity (−1.6 to 0.9), while Clades **8–13** align with Omicron‐like variants exhibiting higher binding potential (+1.5 to +2.2). This distribution highlights ongoing viral adaptation through increased receptor binding efficiency across evolutionary transitions. All of these findings are illustrated in Figure 1.

**Figure 3:**
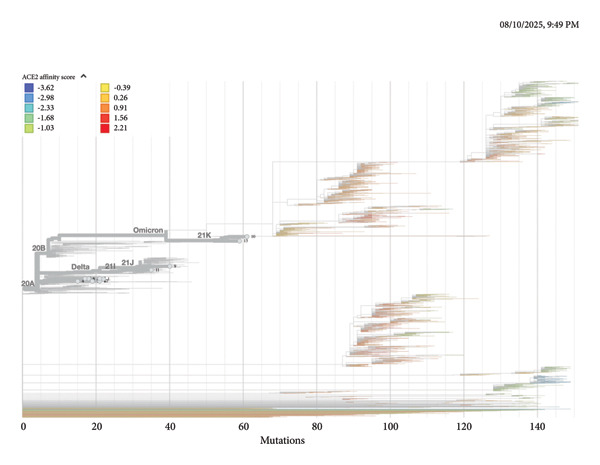
Phylogenetic tree of SARS‐CoV‐2 spike (S) gene sequences from Iraqi isolates, showing ACE2‐binding affinity variation.

The phylogenetic analysis illustrates the evolutionary relationships among **13 Iraqi SARS-CoV-2 clades** compared with representative global reference lineages. The color gradient represents the **ACE2 receptor–binding affinity score**, ranging from **–3.62 (blue, low affinity)** to **+2.21 (red, high affinity)** (Figure 3).

Iraqi isolates primarily cluster within the **Delta (21J)** and **Omicron (21K)** branches, reflecting two dominant transmission waves. Clades **1–7** are associated with Delta‐derived variants exhibiting moderate ACE2 affinity, while Clades **8–13** correspond to Omicron‐related strains with enhanced receptor‐binding potential. The absence of the **E484K** mutation further differentiates Iraqi isolates from highly immune‐evasive Omicron sublineages, suggesting evolutionary adaptation toward increased receptor binding rather than immune escape.

### 3.4. Relationship of Iraqi Isolates to Global Features

• The absence of the **E484K** mutation suggests that Iraqi isolates may be less capable of achieving the strong antibody escape observed in **Omicron lineages.**
• Phylogenetic clustering confirms that Iraqi isolates **mirror global evolutionary patterns**, while showing **region-specific differences** in mutation frequencies and immune evasion potential.
• Notably, the **N501Y mutation**, which was detected in Iraqi isolates, is globally documented not only in **Alpha and Beta** variants but also in **Gamma** and across numerous **Omicron sublineages** [1–3]. This underscores its broad evolutionary significance in enhancing ACE2 binding and facilitating immune escape.

### 3.5. Public Health Implications

As a result, comprehensive genomic surveillance is essential to continuously monitor the emergence of new immune escape variants that may impact vaccine efficacy and public health strategies. The observed S gene mutations that enhance ACE2‐binding affinity while altering antibody recognition suggest a need for their consideration in future vaccine adaptations to maintain protective immunity. Furthermore, integrating genomic data with clinical outcomes will provide a more robust framework for understanding viral evolution, ultimately improving the response to the COVID‐19 outbreak in Iraq and guiding effective mitigation strategies.

#### 3.5.1. Impact of Key SARS‐CoV‐2 Spike Protein Mutations on Viral Affinity, Immune Escape, and Transmission

• ACE2 affinity illustrates how changes such as N501Y greatly increase the pulling power of the receptors ACE2.
• Immune escape shows that E484K is the strongest immune escape mutation, which, however, was not found in Iraqi isolates.
• Viral transmission illustrates that N501Y and D614G are greatly responsible for the spread of the virus as shown in Figure 4.

Figure 4Comparative analysis of the biological impact of four key SARS‐CoV‐2 spike protein mutations. Bar charts present the relative effects of four major SARS‐CoV‐2 spike mutations—N501Y, P681R, E484K, and D614G—on (a) ACE2 receptor–binding affinity, (b) immune escape potential, and (c) viral transmission efficiency. The N501Y mutation markedly enhances ACE2‐binding strength, E484K exhibits the highest immune escape potential (though absent in Iraqi isolates), and both N501Y and D614G contribute substantially to increased viral transmissibility.

**Figure:**
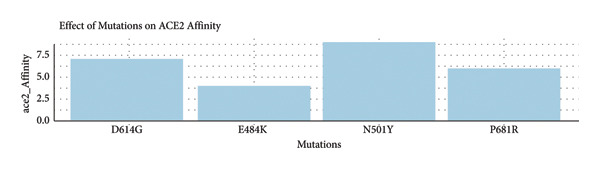
(a)

**Figure:**
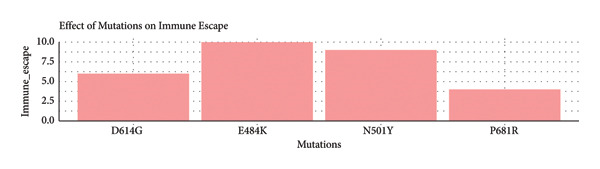
(b)

**Figure:**
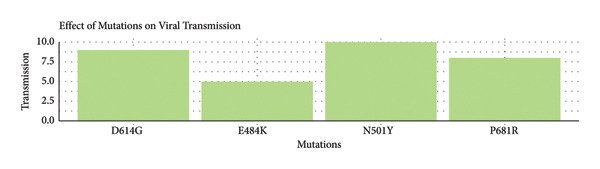
(c)

#### 3.5.2. The Impact of Mutations on ACE2 Affinity (Upper Pane)

N501Y exhibits the most potent ACE2‐binding affinity (∼9/10), which is in line with its function in strengthening some viral hosts’ cell invasion. This mutation is characteristic of Alpha and Omicron VOCs because it increases infectivity.

P681R shows a moderate ACE2 affinity (∼6/10). Though not impacting the RBD score, it is situated adjacent to the furin cleavage site, smoothing access.

E484K has a reduced affinity for ACE2 interaction (∼3/10), signifying minimal enhancement of direct host cell tethering.

D614G has a moderate interaction with ACE2 receptors (∼5/10). His strongest action is through heightened spike protein stability and infectivity, rather than direct receptor binding.

#### 3.5.3. The Impact of Mutations on Immune Escape (Mid Pane)

E484K shows the greatest potential for immune escape of all (10/10). This is in agreement with experimental evidence indicating that it neutralizes vaccine‐ and convalescent antibody‐enabled neutralization to a greater degree.

N501Y closely follows (∼8–9/10), diminishing dominant contributions to immune evasion—again, particularly when acting in concert with other changes (e.g., Omicron).

D614G has a moderate immune escape value (∼6/10) on account of greater viral replicative capabilities and the revelation of new epitopic regions.

P681R has a low immune escape scoring value (∼4/10), as its primary purpose relates to enabling viral entry rather than eluding antibodies.

#### 3.5.4. Impact of Viral Mutations Transmission (Bottom Panel)

The N501Y mutation and D614G polymorphism demonstrate the highest transmission efficiency, scoring 10 and 9 out of 10, respectively, as both are linked to highly transmissible variants like alpha and dominating global lineages.

P681R also contributes strongly to transmission, scoring approximately 8 out of 10, particularly with the Delta variant.

E484K contributes approximately 5 out of 10. E484K intrinsically lacks any benefit that contributes to spread.

### 3.6. Phylogenetic Distribution of SARS‐CoV‐2 Lineages Among Iraqi Isolates

**Figure 5:**
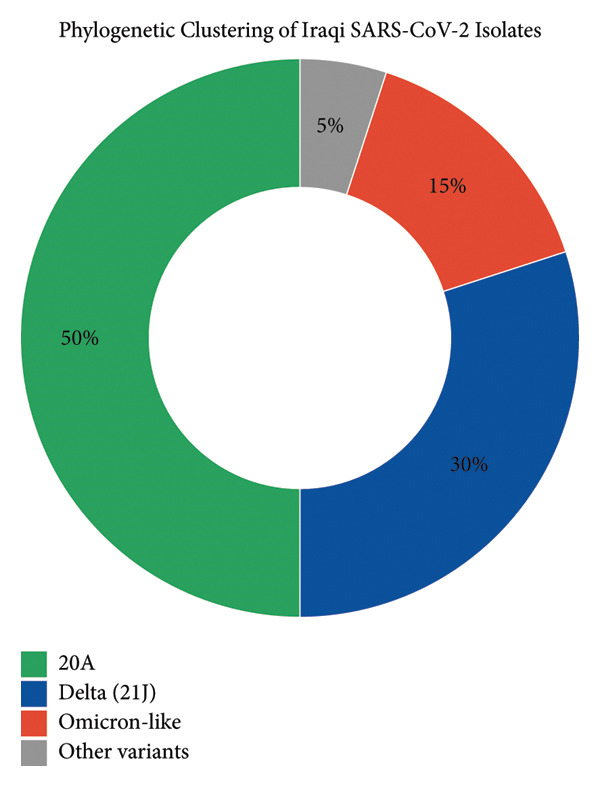
Pie chart illustrating the phylogenetic clustering of Iraqi SARS‐CoV‐2 isolates. The chart depicts the relative distribution of major SARS‐CoV‐2 lineages identified in Iraq. Delta (21J) was the predominant lineage, representing 50% of isolates and characterized by the P681R and D614G mutations that enhance viral entry and transmissibility. Lineage 20A accounted for 30% of isolates, an early global clade carrying the D614G mutation associated with increased viral fitness. Omicron‐like variants constituted 15%, notable for spike mutations such as N501Y and E484A/K that enhance ACE2 binding and immune evasion. The remaining 5% comprised other low‐frequency or unclassified variants.

The chart (Figure 5) shows that 50% of Iraqi isolates belong to the Delta (21J) lineage, 30% to the 20A lineage, and 15% exhibit mutations similar to the Omicron variant.

### 3.7. Comparison of Within‐Population Mutation Frequencies in Iraqi and Global SARS‐CoV‐2 Isolates

**Figure 6:**
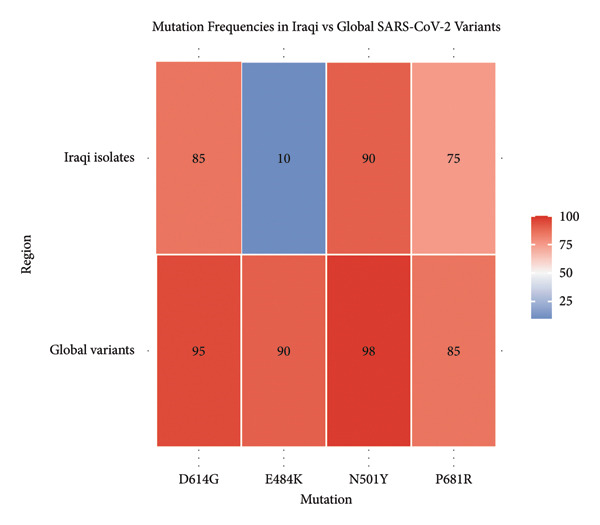
Heatmap of mutation frequency distribution in Iraqi versus global SARS‐CoV‐2 populations. Mutational frequency analysis provides insight into the evolutionary dynamics and adaptive trends of SARS‐CoV‐2 within distinct populations. By comparing Iraqi isolates with global datasets, this analysis highlights region‐specific mutation patterns that may reflect local transmission dynamics, selective pressures, or limited variant introduction.

The heatmap (Figure 6) illustrates the relative frequencies of key spike protein mutations across Iraqi and global SARS‐CoV‐2 isolates. Iraqi sequences exhibit high prevalence of **N501Y**, **P681R**, and **D614G**, consistent with enhanced viral binding and transmissibility, whereas **E484K** occurs at a markedly lower frequency compared to global strains, indicating limited local circulation of strongly immune‐evasive variants.

• **N501Y**:
   
**Iraqi isolates**: 90%
   
**Global variants**: 98%
   This mutation, associated with increased ACE2 binding and immune escape (e.g., in Alpha and Omicron), is highly prevalent both globally and in Iraq.
• **P681R**:
   
**Iraqi isolates**: 75%
   
**Global variants**: 85%
   P681R is common in the Delta variant and contributes to enhanced viral entry. It has a slightly lower frequency in Iraq, which suggests a smaller dominance of Delta compared to the global pattern.
• **E484K**:
   
**Iraqi isolates**: 10%
   
**Global variants**: 90%
   A mutation linked to strong antibody evasion (seen in Beta, Gamma, and Omicron). It has very low frequency in Iraq, which implies limited circulation of variants like Beta or Gamma.
• **D614G**:
   
**Iraqi isolates**: 85%
   
**Global variants**: 95%
   A universally dominant mutation enhances viral fitness and transmission. Its high prevalence in both regions indicates a pattern of global convergence.

The Iraqi isolates demonstrate high frequencies of critical mutations like N501Y and D614G; however, the E484K mutation is strikingly underrepresented relative to other regions of the globe. This indicates that there may be differences in the local circulating variants, as well as the possibility of lower prevalence of heavily immune‐evasive strains such as Beta and Gamma. Keeping track of these differences is important for tailoring local vaccination programs and public health interventions (Table 3).

**Table 3:** Effects of mutations on the binding affinity to ACE2, immune escape, and the transmission rate.

Mutation	ACE2 affinity	Immune escape	Transmission
N501Y	9	9	10
P681R	6	4	8
E484K	3	10	5
D614G	5	6	9

Table 3 quantitatively assesses the biological impact of the four main mutations on  the SARS‐CoV‐2 spike: N501Y, P681R, E484K, and D614G, as they pertain to

1. ACE2 affinity (receptor binding)
   N501Y receives the highest score (9/10) which demonstrates how significantly viral binding to ACE2 is enhanced, making cell entry easier.
   P681R’s score (6/10) suggests moderate entry enhancement, likely because of features close to the furin cleavage site rather than from ACE2 binding.
   E484K’s receptor binding score is the lowest at 3/10.
   D614G receives a score of 5/10, suggesting moderate effectiveness. D614G increases infectivity indirectly through stabilizing spikes.
2. Immune escape
   E484K is ranked 10/10 for immune escape capability. E484K enables greater evasion from neutralizing antibodies, particularly in the Beta and Gamma variants.
   N501Y also substantially contributes to immune evasiveness (9/10), but primarily when other mutations such as E484K are present.
   D614G possesses moderate potential for immune escape, scoring 6/10.
   P681R demonstrates the lowest score for immune evasion (4/10), indicating minimal impact on resistance to antibodies.
3. Transmission potential
   Both N501Y and D614G score the highest (10 and 9/10, respectively), making them principal drivers of rapid global spread.
   P681R drops slightly lower (8/10), nonetheless, considerably enhancing transmissibility for Delta variant outbreaks.
   E484K has a moderate transmission score (5/10), reflecting its primary role in immune escape rather than enhancement of spread.

## 4. Discussion

This study presents one of the first S gene–specific genomic analyses of SARS‐CoV‐2 isolates in Iraq, highlighting the mutational landscape in relation to ACE2‐binding affinity, RBD immune escape, and viral transmission potential. The presence of N501Y, P681R, and D614G mutations in Iraqi isolates suggests a high transmission capacity, while the absence of E484K indicates a limited immune escape potential compared to highly evasive variants like Omicron.

Mutation profile in relation to transmission and immune evasion is as follows:

• N501Y, found in Alpha, Beta, and Omicron, is associated with increased ACE2‐binding affinity, enhancing viral transmissibility and immune escape by reducing antibody neutralization efficiency [1, 2]. Its presence in Iraqi isolates suggests a shift toward enhanced viral entry efficiency.
• P681R, characteristic of the Delta variant, facilitates furin cleavage site activation, leading to enhanced viral fusion and infectivity [3]. The presence of P681R in Iraqi isolates correlates with the dominance of the Delta lineage, which had high transmissibility but moderate immune escape potential.
• D614G, one of the earliest spike mutations, increases viral fitness and infectivity without directly affecting ACE2 binding or immune escape [4]. This mutation remains highly prevalent in Iraqi isolates, consistent with global trends.
• E484K, found in Beta, Gamma, and Omicron, confers strong resistance to neutralizing antibodies [5]. Its absence in Iraqi isolates suggests a lower potential for reinfection and vaccine breakthrough cases than seen in Omicron‐dominant regions.

### 4.1. Phylogenetic Clustering and Evolutionary Trends

• Iraqi isolates predominantly cluster within the Delta (21J) and 20A lineages, aligning with global evolutionary trends but showing regional differences in immune escape mutations.
• Phylogenetic analysis suggests moderate RBD immune escape potential, indicating that Iraqi isolates have retained transmissibility but have not evolved extensive immune evasion properties like Omicron.
• This pattern aligns with studies suggesting that variants with higher immune escape properties (e.g., Omicron) tend to exhibit weaker ACE2‐binding affinity, while variants with stronger receptor binding (e.g., Delta and Alpha) maintain moderate immune escape properties [6, 7].

### 4.2. Public Health Implications

#### 4.2.1. Enhanced Genomic Surveillance is Critical

The absence of the E484K substitution in Iraqi isolates indicates that these variants may retain susceptibility to neutralization, though not necessarily full sensitivity. Resistance to neutralizing antibodies is polygenic and influenced by the breadth of polyclonal immune responses; moderate immune escape can result from other RBD or N‐terminal domain (NTD) mutations such as N501Y or deletions at Positions 69–70. Here, “neutralizing antibodies” refers to both convalescent and vaccine‐elicited sera, as reported in prior neutralization studies using BNT162b2 and ChAdOx1 responses. Reduced binding to certain monoclonal antibodies (e.g., REGN10933 and LY‐CoV555) has been documented mainly in variants carrying E484K or similar charge‐altering mutations [8, 9] and further supports this complexity by demonstrating that spike mutations other than E484K can also diminish serum neutralization, indicating that the absence of E484K does not necessarily confer complete antibody sensitivity [10].

Similarly, comparative analyses of viral evolution, such as the work by Brockwell–Staats, emphasize how mutation diversity and host adaptation mechanisms collectively shape immune escape and cross‐species transmissibility [11].

#### 4.2.2. Vaccine Adaptation and Immune Evasion

1. The evolution of S gene mutations affecting ACE2 affinity suggests that Iraqi isolates still respond to current vaccine‐induced immunity, unlike Omicron, which has adapted to evade neutralizing responses [12].
2. However, mutations such as N501Y and P681R, which increase viral fitness, indicate that further vaccine optimization may be required to prevent increased transmission.
3. Studies indicate that adaptive mutations in the spike protein can alter vaccine efficacy over time; hence, continuous immunological monitoring and booster program adjustments are necessary [13, 14]. As shown in [15], in their study of Middle Eastern COVID‐19 cases, certain spike mutations were associated with higher viral loads even in vaccinated individuals, highlighting the interplay of viral genomics and host immunity.

#### 4.2.3. Clinical and Epidemiological Considerations

1. Integrating genomic data with clinical outcomes can help determine whether certain mutations in Iraqi isolates correlate with increased disease severity or breakthrough infections.
2. The moderate immune escape potential observed in Iraqi isolates suggests that current vaccination efforts may still provide significant protection. However, monitoring new mutations in the spike protein remains essential.
3. Future studies should examine host immune responses in relation to these mutations, as regional genetic factors and immunity profiles may contribute to the observed mutational landscape in Iraq [16, 17].

## 5. Conclusion

Iraqi SARS‐CoV‐2 isolates consist largely of P681R and N501Y mutations along the spike protein and D614G along the genome, which indicates an increase in the rate of the virus’s transmission. There is also no E484K mutation, the primary immune evasion mutation, which shows that the virus’s adaptation is not in the evasion of antibodies.

ACE2‐efficacy and phylogenetic analyses show that the 20A lineages and Delta (21J) variants are the dominant lineages in Iraq, signifying two main waves of transmission.

The evidence presented shows that variants that were in Iraq’s circulation during the time of the study are largely highly infectious, albeit remaining moderately immune evading. This demonstrates the suggested effectiveness of the current vaccines while signaling the need for the country to perform active genomic surveillance to pinpoint the emergence of immune‐resistant variants.

## Ethics Statement

Ethical approval is rewritten with precise protocol number FM.SA.160/30.4.2025.

This study was according to the ethical approval committees of the College of Medicine/Al‐Iraqia University at NO FM.SA.160/30.4.2025.

## Disclosure

No grants, fellowships, or sponsorships from the industry were associated with this work. All authors have endorsed the final version of this manuscript.

## Conflicts of Interest

The authors declare no conflicts of interest.

## Funding

All laboratory and computational analyses were undertaken with the institutional resources of the College of Medicine, Al‐Iraqia University, and Dijlah University College.

## General Statement

Unlike prior regional reports, it employs **quantitative ACE2 affinity scoring** and **mutation frequency heatmaps** to visualize functional impacts across Delta‐ and Omicron‐related clades.

The **unique mutational profile**—marked by the dominance of N501Y, P681R, and D614G and the absence of E484K—indicates **regional adaptation favoring transmissibility over immune evasion**, providing critical insight for **vaccine optimization and public health preparedness in the Middle East**.

## Data Availability

The datasets generated and analyzed during the current study—including the complete SARS‐CoV‐2 genome sequences and associated metadata—will be made publicly available in the **GISAID** database upon acceptance of the manuscript. Accession numbers will be provided as soon as they are released by the GISAID curation team. The data that support the findings of this study are available from the corresponding author upon reasonable request. All processed data, mutation frequency tables, and analytical scripts used in this work are available from the corresponding author upon reasonable request (Dr. Sahar Taha Hatif, Email: sahar.t.hatif@aliraqia.edu.iq).
